# Functional Characterization of Flavanone 3-Hydroxylase (F3H) and Its Role in Anthocyanin and Flavonoid Biosynthesis in Mulberry

**DOI:** 10.3390/molecules27103341

**Published:** 2022-05-23

**Authors:** Mingjie Dai, Xiaoru Kang, Yuqiong Wang, Shuai Huang, Yangyang Guo, Rufeng Wang, Nan Chao, Li Liu

**Affiliations:** 1 College of Biotechnology, Jiangsu University of Science and Technology, Zhenjiang 212018, China; 192310030@stu.just.edu.cn (M.D.); 209310036@stu.just.edu.cn (X.K.); 192211802211@stu.just.edu.cn (Y.W.); 202310006@stu.just.edu.cn (S.H.); 212211831122@stu.just.edu.cn (Y.G.); wangrf@gempharmatech.com (R.W.); 2 Sericultural Research Institute, Chinese Academy of Agricultural Sciences, Zhenjiang 212018, China

**Keywords:** anthocyanidins, flavanone 3-hydroxylase, flavonoid, function, *Morus*

## Abstract

Mulberry (*Morus* spp., Moraceae) is an important economic crop plant and is rich in flavonoids and anthocyanidins in ripe fruits. Anthocyanins are glycosides of anthocyanidins. Flavanone 3-hydroxylase (F3H) catalyzes the conversion of naringenin into dihydroflavonols and is responsible for the biosynthesis of flavonols and anthocyanidins. In this study, *MazsF3H* was cloned and characterized from *Morus atropurpurea* var. *Zhongshen 1.* Conserved motif analysis based on alignment and phylogenetic analysis indicated that *MazsF3H* belonged to 2-oxoglutarate-dependent dioxygenase and *MazsF3H* clustered with F3Hs from other plants. *MazsF3H* was located in both nucleus and cytosol. *MazsF3H* was expressed in stems, leaves, stigmas and ovaries, except buds. *F3H* expression levels showed a positive and close relationship with anthocyanin content during the anthocyanin-rich fruit ripening process, while it showed a negative correlation with anthocyanin content in *LvShenZi*, whose fruits are white and would not experience anthocyanin accumulation during fruit ripening. Significantly different *F3H* expression levels were also found in different mulberry varieties that have quite different anthocyanin contents in ripe fruits. Overexpression *MazsF3H* in tobacco showed unexpected results, including decreased anthocyanin content. Down-regulation of *F3H* expression levels resulted in co-expression of the genes involved in anthocyanin biosynthesis and a significant decrease in anthocyanin content, but the change in total flavonoid content was subtle. Our results indicated that F3H may play quite different roles in different varieties that have quite different fruit colors. In addition, possible complex regulation of flavonoid biosynthesis should be further explored in some of the featured plant species.

## 1. Introduction

The flavonoid biosynthetic pathway has been well investigated and is thought to be a gold mine for metabolic engineering [1]. The application of biological engineering of flavonoids is prevalent, and there have been many studies on the modification of color, the change in medicinal plant composition, and the improvement of crop nutrition. The flavonoid class of secondary metabolites mainly contains anthocyanins, flavonols, flavones and proanthocyanidins [2,3]. Anthocyanin is essential to the physiological and biochemical activities of plants, including antioxidation, protection of plant damage, stress responses, and attracting animals to pollinate and spread seeds [4]. In recent years, more and more attention has been paid to anthocyanins because of their prominent role in medical resources [5].

Flavonoid-related genes are primarily responsible for flavonoid biosynthesis, and the activities of different biosynthesis enzymes control the flavonoid profile [6]. Flavonoid biosynthesis starts with the general phenylpropanoid pathway, producing 4-coumaroyl-CoA by phenylalanine ammonia lyase (PAL) and cinnamate-4-hydroxylase (C4H). 4-coumaroyl-CoA as precursor enters the flavonoid biosynthesis pathway, leading to different metabolic branches, including the products of chalcones, aurones, isoflavonoids, flavones, flavonols, flavandiols, anthocyanins [7]. This first committed step in flavonoid biosynthesis is catalyzed by chalcone synthase (CHS), which uses malonyl CoA and 4-coumaroyl CoA as substrates [8]. The product naringenin chalcone is subsequently catalyzed by chalcone isomerase (CHI), and the product naringenin is a general precursor of flavonols, anthocyanins, proanthocyanidins, flavones, and isoflavones [9]. Favone synthase I (FNS I) or flavone synthase II (FNS II) participate in the synthesis of flavones from naringenin. Isoflavone biosynthesis starts from naringenin or liquirtigenin, in a reaction catalyzed by Isoflavone synthase (IFS), as the first step [10]. Naringenin is also converted to dihydroflavonols by flavanone 3-hydroxylase (F3H). The dihydroflavonols are precursors of flavonols and anthocyanins. Flavonol synthase (*FLS*) is responsible for the synthesis of flavonols and dihydroflavonol reductase (*DFR*), flavonoid 3′-hydroxylase(F3′H), anthocyanidin synthase (ANS), and UDP-glucose: flavonoid 3-glucosyltransferase (UFGT) are responsible for the subsequent biosynthesis of anthocyanins [10].

The flavonoid pathway-related enzymes F3H, FLS and ANS all belong to the 2-oxoglutarate-dependent dioxygenase (2-ODD) subfamily [11,12]. F3H is a key enzyme in directing carbon flow towards the biosynthesis of 3-hydroxylated flavonoids and is responsible for the biosynthesis of flavonols and anthocyanidins [13]. Down-regulation of *F3H* in strawberry resulted in a great decrease in anthocyanin and a moderate decrease in flavonol content [14]. Overexpression of *Lycium chinense LcF3H* in tobacco showed increased content of flavan-3-ols and increased tolerance to drought stress [15]. However, overexpression of *Sorghum bicolor SbF3H* resulted in a subtle impact on flavonol production in tissues with endogenous F3H activities [13]. *Carthamus tinctorius CtF3H* showed diametrically opposite expression patterns in different phenotypes with orange-yellow flowers and white flowers when exposed to external methyl jasmonate (MeJA) treatment, which has been identified as an elicitor of flavonoid metabolites [16]. The above facts suggest that the function of F3H in flavonoid biosynthesis is highly complex, and that its impact on flavonoid profile might be species-specific or phenotype-specific.

Mulberry (*Morus* spp., Moraceae) plants are distributed widely in China and East Asia and are known as important economic plants with nutritional, medicinal, and ecological value [17,18]. Mulberry fruits are a nutritional foodstuff and are rich in flavonoids and anthocyanins. They are recommended as having antioxidant, antimicrobial, and anti-inflammatory properties [18]. Previous studies have reported that expression levels of genes involved in anthocyanin biosynthesis, including *CHS1*, *CHI*, *F3H1*, *F3′H1*, and *ANS* showed a close correlation with anthocyanin content during the fruit ripening process [19,20]. Given the complex roles played by *F3H* in flavonoid and anthocyanin biosynthesis, it is still necessary to further elucidate how *F3H* functions in the flavonoid biosynthesis pathway and anthocyanin accumulation during fruit ripening in mulberry. In the present study, we functionally characterized *F3H* in mulberry and found its variety-specific impact on anthocyanin accumulation. The expression patterns of *F3H* differ greatly among different mulberry varieties during the fruit ripening process. Generally, down-regulation of *F3H* in mulberry can result in a decrease in anthocyanins and a subtle increase in flavonoid content, while overexpression of mulberry *F3H* in tobacco also resulted in a significant decrease in anthocyanins. This suggests that more complex regulation mechanisms of flavonoid profile partitioning may possibly exist in mulberry.

## 2. Results

### 2.1. Molecular Cloning and Characterization of MazsF3H in Mulberry

**Figure 1:**
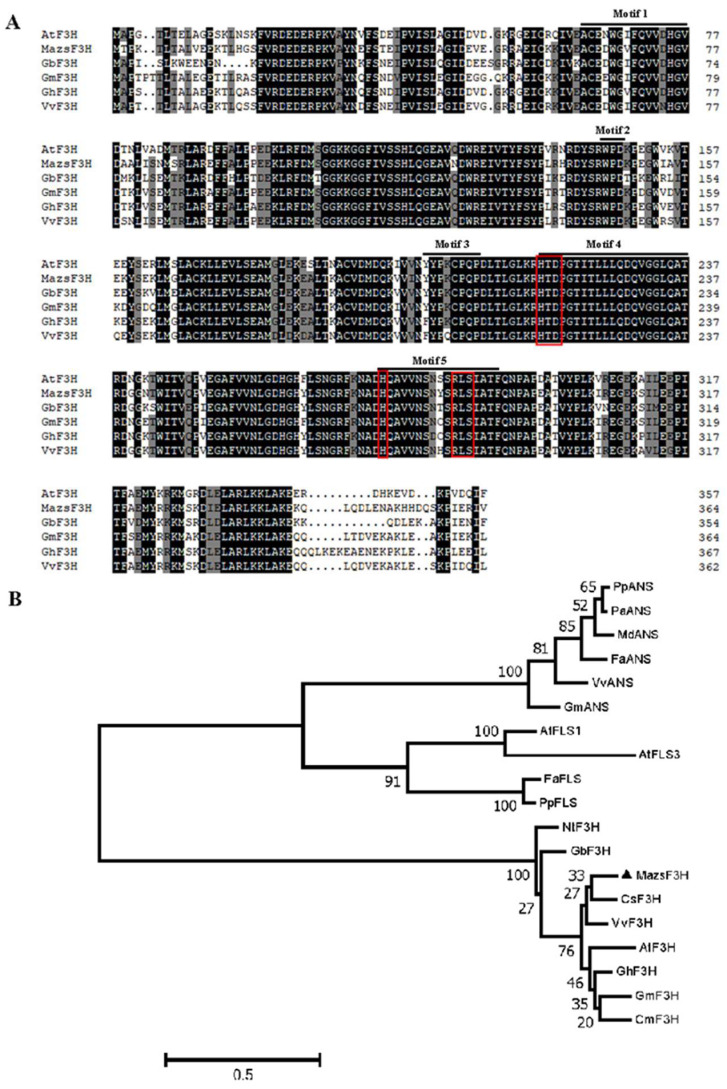
Alignment and phylogenetic analysis of F3Hs: (**A**) alignment of mulberry *MazsF3H* and other F3Hs from different plants. Red boxes indicate the ferrous iron ligation motif HXDX55H and a 2oxoglutarate (2-ODD) binding motif RXS (RLS). Five conserved motifs in plant 2-oxoglutarate-dependent dioxygenase were also marked; (**B**) maximum-likelihood tree was constructed using JTT + G model and 1000 bootstrap replicates were selected for evaluate the reliability of tree, *MazsF3H* was indicated full triangle. All sequences used for alignment and phylogenetic analysis are listed in Table S1.

**Figure 2:**
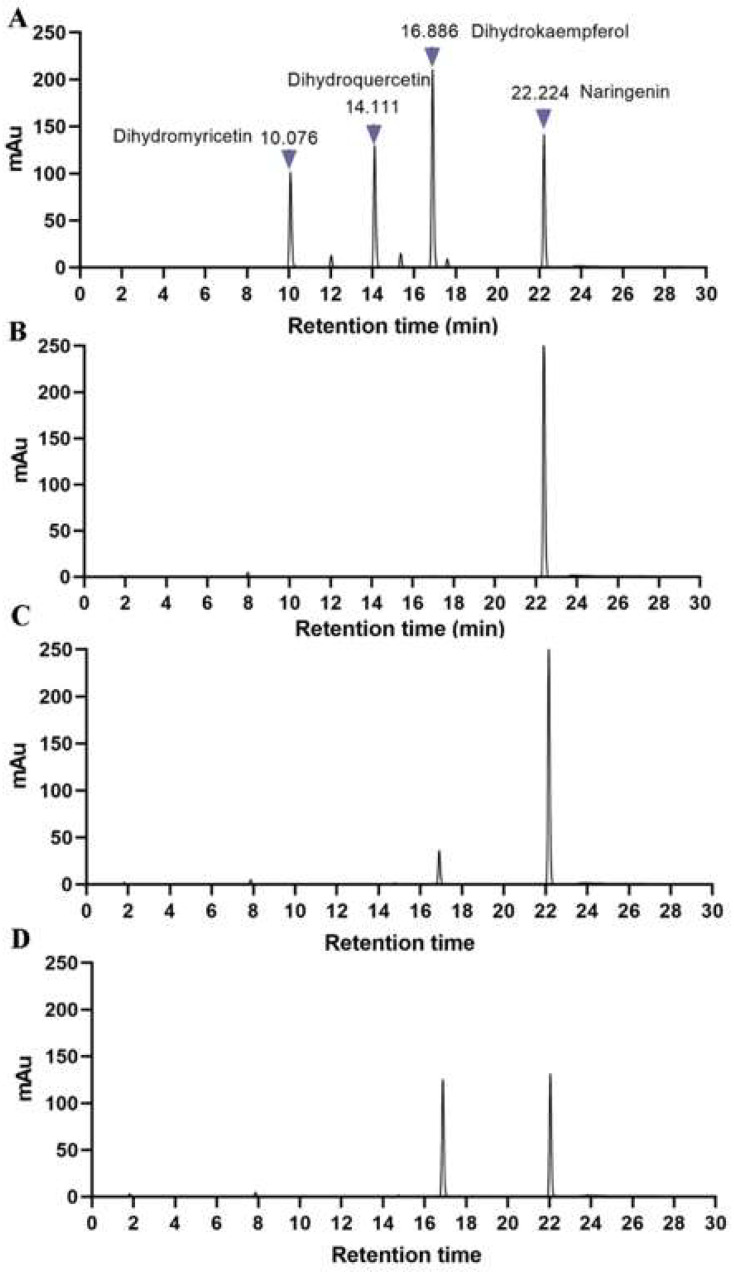
Enzymatic assay of *MazsF3H* in vitro using HPLC-VWD: (**A**) Chromatogram of standards. (**B**) Reaction containing boiled *MazsF3H* as control. (**C**) Reaction containing 10 μg *MazsF3H* proteins. (**D**) Reaction containing 20 μg *MazsF3H* proteins.

An *F3H* gene with 1098 bp CDS (coding sequence) coding 365 amino acids, named *MazsF3H*, was successfully cloned in *Morus atropurpurea* var. *Zhongshen 1. MazsF3H* has been deposited in NCBI (Accession number: ON055162). The weight of the putative protein coded by *MazsF3H* is about 42 kDa. The alignment of *MazsF3H* with other functionally characterized F3Hs showed that high sequence identity (86.5% identity) could be observed among these F3Hs. Conserved motifs of 2-oxoglutarate-dependent dioxygenase, including the ferrous iron ligation motif HXDX55H and the 2oxoglutarate (2-ODD) binding motif RXS (RLS), were detected in *MazsF3H* (Figure 1A). In addition, five commonly conserved motifs in plant 2-oxoglutarate-dependent dioxygenase were also indicated and were found in *MazsF3H*. Phylogenetic analysis of 2-oxoglutarate-dependent dioxygenases in plants showed that *MazsF3H* clustered with other F3Hs, including *Vitis vinifera* (Vv) F3H, *Arabidopsis thaliana* (At) F3H et al., and can be distinguished from FLSs and ANSs (Figure 1B). Enzymatic assay of purified *MazsF3H* in vitro showed that *MazsF3H* can convert naringenins to dihydrokaempferols (Figure 2).

### 2.2. Subcellular Location of MazsF3H

**Figure 3:**
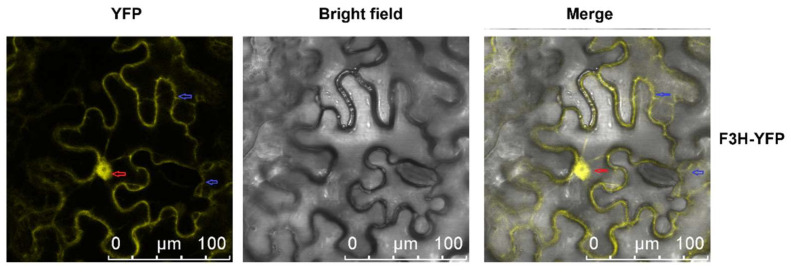
Subcellular location of *MazsF3H*. The red arrow indicates the nucleus and the blue arrow indicates the cytosol.

Subcellular location of *MazsF3H* was indicated by yellow fluorescent protein (YFP) and showed that *MazsF3H* was located in both the cytosol and nucleus (Figure 3). The cytosol and nucleus locations of structure genes involved in flavonoid biosynthesis pathway has been reported in several plants and is supposed to correspond with the in situ biosynthesis of flavonoids in the cytosol and the nucleus [21,22].

### 2.3. Expression Profile of F3H and Its Relationship with the Accumulation of Anthocyanins

**Figure 4:**
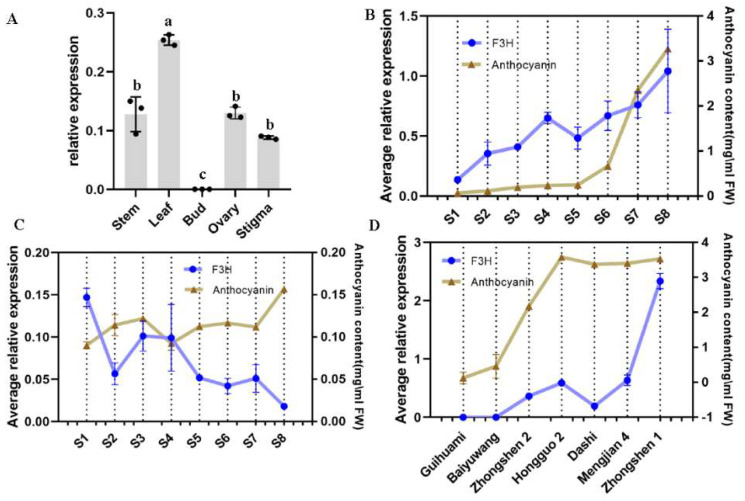
*F3H* expression profiles and anthocyanin content in mulberry: (**A**) Expression profiles of *F3H* in different organs in mulberry. (**B**) *F3H* expression level and anthocyanin content during fruit ripening process in *Zhongshen 1.* (**C**) *F3H* expression level and anthocyanin content during fruit ripening process in fruits at eight different development stages in *LvShenZi.* (**D**) *F3H* expression levels in ripe fruits of different mulberry varieties. The significance is indicated by different letters (*p* < 0.05).

*MazsF3H* was expressed in all detected organs except buds and showed the highest expression level in leaves. Moderate expression levels were observed in stems, ovaries, and stigmata (Figure 4A). To further study the relationship between *F3H* expression level and anthocyanin content during fruit ripening, both *F3H* expression levels and anthocyanin content of mulberry fruit at different growth stages were detected in two mulberry varieties: *Zhongshen 1* (purple fruit) and *LvShenZi* (white fruit) (Figure 4C). Quite different expression patterns of *F3H* were observed in *Zhongshen1* and *LvShenZi*. In *Zhongshen 1*, which is rich in anthocyanins in ripe fruit, *F3H* showed increased expression level along with the ripening process and had a significant positive correlation with anthocyanin accumulation (Figure 4B and Table 1). Meanwhile, in *LvShenZi*, in which there was no anthocyanin accumulation during fruit ripening, *F3H* showed a significant negative correlation with anthocyanin content during fruit ripening. Further study on *F3H* expression levels in ripe fruits from different mulberry varieties were also performed, and the results showed that *F3H* had obvious high expression levels in anthocyanin-rich fruit, but without significant correlation with anthocyanin accumulation (Figure 4D and Table 1). The above results suggest that *F3H* may play different roles in mulberry varieties that have quite different anthocyanin contents, and that the regulation of anthocyanin content is more complex.

### 2.4. Overexpression of MazsF3H in Tobacco Resulting in Change in Anthocyanin Content

**Figure 5:**
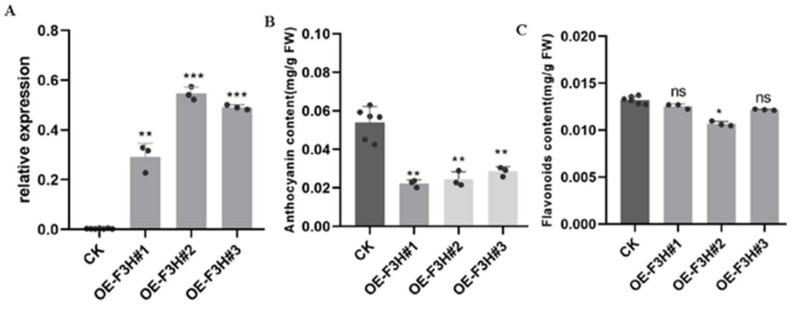
Overexpression of *MazsF3H* in tobacco: (**A**) Expression levels of *MazsF3H* in overexpression transgenic tobacco plants. (**B**) Anthocyanin content in overexpression transgenic tobacco plants. (**C**) Flavonoid content in overexpression transgenic tobacco plants. The significance is marked using * (0.01 < *p* < 0.05), ** (0.001 < *p* < 0.01), *** (*p* < 0.001).

Transient overexpression of *MazsF3H* in tobacco was performed, and three transgenic tobacco lines *OE-F3H#1*, *#2*, *#3* were obtained and confirmed by qRT-PCR, indicating the significant overexpression of *MazsF3H* (Figure 5A). However, the anthocyanin content in the overexpression tobacco leaves showed a significant decrease, while the total flavonoids showed no significant change except in *OE-F3H#2*, which had decreased flavonoid content (Figure 5B,C).

### 2.5. Down-Regulation of F3H Resulting in Decreased Anthocyanin Content and Increased Total Flavonoid Content in Mulberry

**Figure 6:**
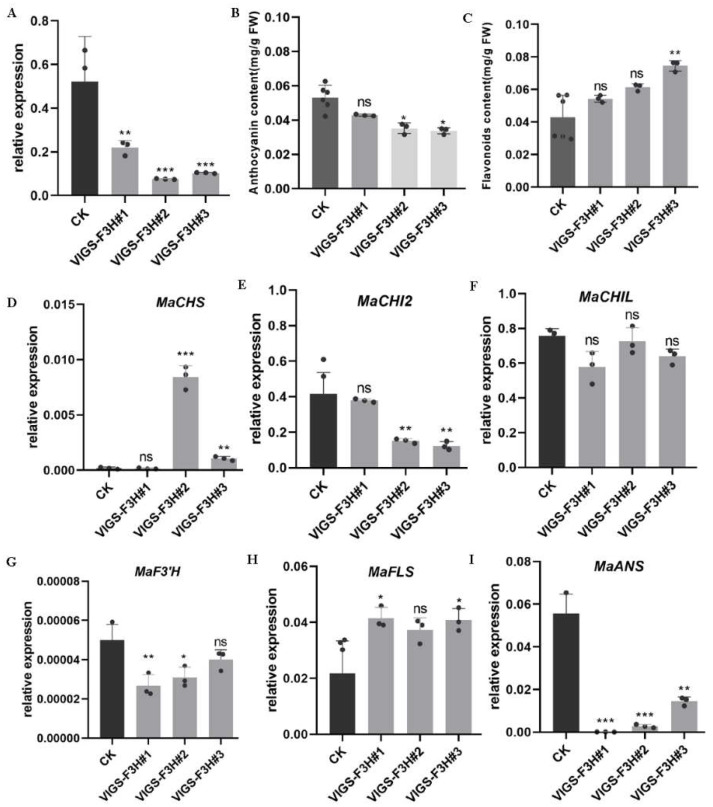
Down-regulation of *F3H* in mulberry: (**A**) Expression levels of *F3H* in VIGS transgenic mulberry plants. (**B**) Anthocyanin content in VIGS transgenic mulberry plants. (**C**) Flavonoid content in VIGS transgenic mulberry plants. (**D**–**I**) The expression levels of genes involved in flavonoid biosynthesis in VIGS transgenic mulberry plants. (* *p* < 0.05, ** *p* < 0.01, *** *p* < 0.001).

qRT-PCR for *F3H* was performed to determine the knock-down efficiency of each positive transgenic mulberry. Expression levels of *F3H* decreased by about 61.5–75.2% in transgenic mulberry plants injected with *pTRV2-MazsF3H* compared to that in CK (Figure 6A). The anthocyanin and total flavonoid contents exhibited no significant change in *VIGS-F3H#1*, which suffered a 61.5% decrease in *F3H* expression level. The anthocyanin contents were significantly decreased in *VIGS-F3H#2* (75.2% decrease in *F3H* expression level) and *VIGS-F3H#3* (70.1% decrease in *F3H* expression level) (Figure 6B,C). This suggests that severe decrease in *F3H* expression level can result in a significant decrease in anthocyanins.

The expression changes in structure genes involved in the flavonoid pathway were also determined when *F3H* was knocked-down. The genes involved in flavonoid precursor biosynthesis, including *CHS*, *CHI2* and *CHIL (CHI-like,* IV class *CHI)*, showed quite different changes. *CHS* showed increased expression, while *CHI2* showed decreased expression levels in mulberry plants, with >70% decrease in *F3H* expression, while there was no significant change in *VIGS-F3H#1* (Figure 6D,E). *CHIL*, which has been reported to be a *CHS* interaction protein and to have a role in enhancing flavonoid biosynthesis, showed no significant change (Figure 6F). The genes involved in anthocyanin biosynthesis, including *F3′H* and *ANS*, were significantly decreased (Figure 6G,I). *FLS*, which is responsible for flavonol biosynthesis, showed significantly increased expression levels in *F3H* down-regulated mulberry (Figure 6H). The above results show that down-stream anthocyanin biosynthesis-related genes *F3′H* and *ANS* are co-expressed with *F3H* and could be responsible for anthocyanin accumulation, while genes involved in the biosynthesis of different flavonoid profiles have different responses to the disturbance of *F3H* expression levels.

## 3. Discussion

Many previous studies have reported the relationships between structure genes involved in flavonoid biosynthesis and the accumulation of flavonoids and anthocyanins [19,23,24]. In mulberry, *CHS1*, *CHI*, *F3H1*, *F3′H1* (flavonoid 3′-hydroxylase), and *ANS* have been reported to correlate with anthocyanin biosynthesis during the fruit ripening process [19]. Our previous study on *MmCHI1* and *MmCHI2* from *Morus multicaulis* also showed that the dominant *CHI MaCHI2* had a positive correlation with anthocyanin accumulation in fruit [25]. The present study shows that *F3H* expression levels have a positive correlation with anthocyanin accumulation in anthocyanin-rich mulberry fruits, while a negative correlation was found with anthocyanin content in mulberry fruits that did not require anthocyanin accumulation during fruit ripening process. These results indicate that different mechanisms may exist for regulation of flavonoid or anthocyanin biosynthesis in different mulberry varieties.

The subcellular locations of enzymes involved in secondary metabolite biosynthesis affect the utility of intermediate products and the efficiency of biosynthesis. Enzymes involved in the biosynthesis of secondary metabolites can form weakly bound, ordered complexes, which are referred to as “metabolons” [26]. Lignins and flavonoids are products of the phenylpropanoid pathway, and both lignin and flavonoid metabolons have been reported in many plants [26,27]. Flavonoid metabolons appear to be organized as multi-enzyme complexes, mainly at the endoplasmic reticulum (ER) and exclusively in cytoplasm [21]. However, flavonoids have been reported to be distributed in different cell compartments, including the cytosol, vacuole, ER, chloroplast, nucleus, and small vesicles, as well as in extracellular space, in different types of cells [21,22]. Despite the transport of flavonoid in cells, in situ biosynthesis of flavonoids in the nucleus has also been reported. Subcellular location analysis showed that some enzymes involved in flavonoid biosynthesis were located in both the nucleus and the cytosol. Arabidopsis AtCHS and AtCHI, CtF3H, Sm(eggplant)CHS, SmCHI, SmDFR, SmANS and *MazsF3H*, in this study, were all located in both the nucleus and the cytosol [21,28,29]. Meanwhile, lignin-related enzymes have been reported to be exclusively located in cytoplasm [30]. Given that flavonoids can bind to proteins and nucleic acids and play important roles in various biological processes, including as UV protectants and modifiers of auxin transport, in situ flavonoid biosynthesis in the nucleus may serve to protect DNA from UV or to control the transcription of genes [21,22,31]. It would be interesting to explore the biosynthesis mechanism of in situ flavonoid biosynthesis in the nucleus and its possible role in transcription regulation.

The regulation of structure genes involved in flavonoid biosynthesis could affect the accumulation of the products. Overexpression of *CHS* and *CHI* can enhance flavonoid and anthocyanin biosynthesis, and down-regulation of *CHI* results in a decrease in anthocyanin content [8,25,32]. The regulation of *F3H* expression may have quite different results in different plants. In addition, *F3H* even exhibited contrastings responses to methyl jasmonate (MeJA) in different plant phenotypes [16]. Our results also demonstrated the complex roles of *F3H* in flavonoid biosynthesis in different mulberry varieties. The expression of late anthocyanin biosynthesis genes (*UFGT*, *ANS*, *DFR* and *CYP75B1*) in fruits was much lower in non-pigmented mulberry, while the ratio of flavonols to flavonoids was higher in non-pigmented mulberry, which indicated the redirected the flux in the flavonoid pathway in non-pigmented mulberry [33]. The relatively higher expression levels of *F3H* in S1-S4 could be responsible for the accumulation of flavonols and the precursors of anthocyanins. During S5-S8, feedback regulation of *F3H* may result in decreased *F3H* expression levels while the accumulation of anthocyanins is still being slowly catalyzed by late anthocyanin biosynthesis enzymes, resulting in the observation of a negative correlation betwen F3H expression level and anthocyanin content. It is necessary to consider the phenotype first when trying to modify *F3H* to regulate flavonoid or anthocyanin biosynthesis. In addition, the multi-level regulation of flavonoid biosynthesis in plants has also been reported, including plant hormones, transcript factors and structure genes, and protein complex. Our previous study suggested that more complex metabolons may exist in mulberry [25]. Flavonoid biosynthesis in plants may be more complex, and more studies still need to be performed to reveal the regulation network covering different levels, especially in some featured plants.

## 4. Materials and Methods

### 4.1. Plant Materials

Samples of leaves, buds, stems, stigmata and ovaries, and fruits from *Zhongshen 1* were collected in March 2021. These samples were used for gene cloning and expression profile analysis. The samples of mulberry fruits at different development stages from *Zhongshen 1* and *LvShenZi* were reported in our previous study [25]. *Morus atropurpurea* variety *Zhongshen 1*(*Mazs*) has ripe fruits that are purple and *Morus alba* var. *LvShenZi* (*LSZ*) has ripe fruits that are white. All of the above samples were collected from the national mulberry germplasm field of the Chinese Academy of Agricultural Sciences, Zhenjiang and immediately stored at −80 °C. At least six mulberry fruits were collected from each mulberry plant. Two-week-old seedlings of *Morus alba* var. *Fengchi* were used to perform virus-induced gene silencing to down-regulation of *F3H*.

### 4.2. Isolation of RNA and cDNA Synthesis

Samples were ground with liquid nitrogen and total RNA was extracted using Plant RN38 Kit (Aidlab, Beijing, China) according to the manual. cDNA was synthesized with the PC54-TRUEscript RT kit (Aidlab, Beijing, China) according to the manufacturer’s protocol.

### 4.3. Cloning of MazsF3H

The *F3H* coding region sequence was extracted from the *morus alba* genome using the annotation file based on the Blast results with *AtF3H* as query. The primers were designed and then synthesized using Sangon Biotech (Shanghai, China). The standard three-step PCR process was adopted with an annealing temperature of 54 °C to amplify *F3H* from *Zhongshen 1*. The PCR products were purified using SanPrep Column DNA Gel Extraction Kit (Sangon Biotech, Shanghai, China) and then cloned into the pMD18-T vector (Takara, Dalian, China). The sequences were deposited at GenBank (Accession number: ON055162) and named *MazsF3H*. Primer information is available in Table S2.

### 4.4. Alignment and Phylogenetic Analysis of F3Hs in Plants

*MazsF3H* and F3Hs from different plants, including *Arabidopsis thialana* AtF3H, *Gynura bicolor* GbF3H, *Garcinia mangostana* GmF3H, *Gossypium hirsutum* GhF3H and *Vitis vinifera* VvF3H, were aligned using DNAman 8.0 (Lynnon BioSoft, QC, Canada) with the default parameters to detect the conserved motifs. In addition, alignment of 2-oxoglutarate-dependent dioxygenases (2-ODDs), including F3Hs, *FLSs* and *ANSs*, was performed to construct the phylogenetic tree. The phylogenetic tree was constructed using Mega 7.0 by means of the maximum-likelihood method using JTT substitution model and the G + I rates among sites model. The tree was assessed using the bootstrapping method with 1000 bootstrap replicates, and marked above nodes only if greater than 50 [34]. 

### 4.5. Prokaryotic Expression and Purification of MazsF3H

Recombinant plasmids pET28a-*MazsF3H* were constructed using seamless cloning (CV1901 kit, Aidlab, Beijing, China) and then confirmed by sequencing. Recombinant plasmids were transferred into *E.coli BL21*(DE3) cells and then incubated at 37 °C in LB media containing kanamycin (50 μg/mL). Finally, a concentration of 0.4 mM isopropy-β-D-thiogalactoside (IPTG) was added at OD = 0.6. Cells were collected by centrifugation at 4000 g after 4 h of incubation at 28 °C. The fusion protein was purified using Ni-NTA Sefinose Resin according to the user’s manual (BBI Life Sciences, Shanghai, China). SDS-PAGE and Bradford Protein Assay Kit (Sangon Biotech, Shanghai, China) were used to detect purified proteins and to determine the concentration of purified proteins.

### 4.6. Enzymatic Assay of MazsF3H

In vitro *MazsF3H* activity was determined as described by Si et al. (2022) with some modifications. The total 400 μL reaction mixture contained 25 mM Tris-HCl (pH 7.4), 0.4 mM naringenin, 0.5 mM dithiothreitol, 1 mM ascorbate acid, 0.2 mM ferrous sulfate, 1 mM 2-oxo-glutarate, and 10 μg or 20 μg recombinant proteins. Sample with protein that had been incubated in boiling water for 10 min were used as the negative control.

The reaction mixture was filtered using 0.22 μm PES membranes before sending them to the HPLC-VWD (Agilent 1260 infinity II, Victoria, TX, USA) system with a Poroshell EC-C18 column (4.6 mm × 150 mm, 4.0 μm, Agilent, Victoria, TX, USA). The mobile phases were methanol (A) and 1% (*v/v*) H3PO4 in water (B) with the following program: 0–20 min, 15% A–−60% A; 20–26 min, 60% A–0%; 26–27 min, 0% A–15% A at a flow rate of 0.8 mL/min. The substrates and products were detected at 290 nm. Standards including naringenin, dihydrokaempferol, dihydroquercetin and dihydromyricetin were purchased from Solarbio (Beijing, China).

### 4.7. Expression Profile of F3H in Mulberry

qRT-PCR (quantitative real-time PCR) was performed to explore the expression of *MazsF3H* in different organs, its expression pattern throughout the fruit ripening process, and in the differences in its expression in ripe fruits of different mulberry varieties using an ABI StepOnePlus™ Real-Time PCR System (Foster City, CA, USA). The primers are presented in Table S2. Actin was used as a reference gene [35]. GraphPad Prism 8.0 was used to visualize the qRT-PCR results and perform ANOVA. *p* < 0.05 was denoted as significance. Firstly, three biological replicates were mixed, and then three technical replicates were performed, respectively, for qRT-PCR.

### 4.8. Subcellular Location of MazsF3H

The method used for the subcellular location of *MazsF3H* was the same as that reported in our previous study [36,37]. The pBI121 vector with *MazsF3H* gene and yellow fluorescent protein (YFP) fusion expression was constructed. The confirmed recombinant plasmids were transferred into *Agrobacterium tumefaciens* strain *GV3101*, which were then transferred into tobacco leaves via Agrobacterium-mediated transient transformation [38]. The YFP fluorescence in leaves was observed using a Leica TCS SP8 confocal microscope (Leica Microsystems, Wetzlar, Germany).

### 4.9. Measurement of Anthocyanin and Total Flavonoid Content

The extraction and measurement of anthocyanin content were performed according to our reported methods, modified from the methods provided by Mehrtens et al. (2005) [39]. The anthocyanin content is given in cyanidin-3-glucoside equivalents. The total flavonoid content was measured with an aluminum nitrate method, using rutin as a reference substance according to a previous study [40].

### 4.10. Transient Overexpression of MazsF3H in Nicotiana Benthamiana

The recombinant plasmids *pNC-1304-35S:MazsF3H* were constructed using nimble cloning [41]. Both recombinant plasmids *pNC-1304-35S:F3H* and empty vector pNC-1304-35S:GFP, as the negative control, were transformed into *Agrobacterium tumefaciens GV3101* and then transferred into *N. benthamiana* leaves via Agrobacterium-mediated transient transformation, as per a previous report [38]. Overexpression of *MazsF3H* was determined using qRT-PCR by comparing the expression levels of target genes in transgenic plants with those in the negative controls.

### 4.11. Obtaining MazsF3H VIGS Transgenic Mulberry

Virus-induced gene silencing (VIGS) was used to obtain *MazsF3H* down-regulated transgenic mulberry, in accordance with our previous report [25,42]. *Agrobacterium tumefaciens* containing recombinant plasmids *pTRV2-MazsF3H*, *pTRV1* and *pTRV2* (negative control) was cultured in transient transformation buffer and then transferred into mulberry leaves by means of pressure injection. Three independent mulberry plants were injected. The knock-down efficiency for the target genes was determined by qRT-PCR 15 days after injection by comparing the transgenic plants with the negative controls and wild types. The correlation analysis and ANOVA analysis were performed using SPSS19.0. 

## 5. Conclusions

In conclusion, our results characterized *MazsF3H* from the *Morus atropurpurea* variety *Zhongshen 1* and revealed that *F3H* may play quite different roles in different varieties that have quite different fruit phenotypes. Transcriptional regulation of *F3H* can result in a quite different impact on the genes involved in flavonoid precursor biosynthesis and the genes involved in anthocyanin biosynthesis, as well as unexpected changes in flavonoid or anthocyanin content. This potential complex regulation of flavonoid biosynthesis should be further explored in some of the featured plant species.

## Figures and Tables

**Table 1:** Correlation of *F3H* expression level with anthocyanin content.

	*F3H* Expression vs. Anthocyanin Content		
	DS-Development	LSZ Development	Varieties
Correlation coefficient	0.8462 **	−0.7449 *	0.569
*p* values	0.0081	0.034	0.1825

The significance is marked using * (0.01 < *p* < 0.05), ** (0.001 < *p* < 0.01).

## Data Availability

The data presented in this study are available in the article and Supplementary Materials.

## Supplementary Materials

The following supporting information can be downloaded at: https://www.mdpi.com/article/10.3390/molecules27103341/s1, Table S1 sequences used for phylogenetic analysis; Table S2 Primers used in this study.
