# Supplementary material for: Functional Characterization of Flavanone 3-Hydroxylase (F3H) and Its Role in Anthocyanin and Flavonoid Biosynthesis in Mulberry
Source: Molecules. 2022 May 23;27(10):3341. doi: 10.3390/molecules27103341 (PMC9144561; doi:10.3390/molecules27103341)
Supplement: Supplementary file 1 [file molecules-27-03341-s001.zip › molecules-1688747-supplementary.pdf]

Table S1 sequences used for phylogenetic analysis.

Symbols	Accession number	Species
AtF3H	NP_190692.1	Arabidopsis thaliana
GbF3H	BAJ17667.1	Gynura bicolor
NtF3H	AAC15414.1	Nicotiana tabacum
GmF3H	ACM62745.1	Garcinia mangostana
GhF3H	ABA01482.1	Gossypium hirsutum
CsF3H	AAT68774.1	Camellia sinensis
CmF3H	ACR77526.1	Citrus maxima
VvF3H	NP_001268034	Vitis vinifera
FaFLS	AAZ78661	Fragaria × ananassa
PpFLS	AJO70134	Prunus persica
AtFLS1	At5g08640	A.thaliana
AtFLS3	At5g63590	A.thaliana
PpANS	AJA79070	Prunus persica
PaANS	ADZ54785	Prunus avium
FaANS	AFK32781	Fragaria × ananassa
VvANS	ABV82967	Vitis vinifera
MdANS	AAD26205	Malus domestica
GmANS	AAR26525.1	Glycine max

Table S2 Primers used in this study.

Primer name	5'-3' sequence	notes
F3H-F	ATGACTCCCAAAACTCTCAC	Cloning
F3H-R	TTAAGCAACAATCCGCTCAA	Cloning
Nib-F3H-F	agtggtctctgtccagtcctATGACTCCCAAAACTCTCAC	Overexpression
Nib-F3H-R	ggtctcagcagaccacaagtTTAAGCAACAATCCGCTCAA	Overexpression
NtActin-QR	GAGGGAAAGAACAGCCTGAATG	qRT-PCR
NtActin-QF	TCACAGAAGCTCCTCCTAATCCA	qRT-PCR
AtActin-QF	GCTCCTCTTAACCCAAAGGC	qRT-PCR
AtActin-QR	CACACCATCACCAGAATCCAGC	qRT-PCR
CHS-QF	TGGTGTAGCCTCAATTCTAGC	qRT-PCR
CHS-QR	CCCACTCTTTGAGGGCCTCTA	qRT-PCR
F3H-QF	CTACAACGAATTCAGCACCGA	qRT-PCR
F3H-QR	GCGGGTACGAGAAGTATGTC	qRT-PCR
ANS-QF	AGGGAGAAATGCAAGGAGGAG	qRT-PCR
ANS-QR	TACTCACTCGTCGCCTCTTT	qRT-PCR
F3'H-QF	ATGGTCATGAGGGTGGCAAG	qRT-PCR

F3'H-QR	CGTCGAGTCTCTGGACTTGG	qRT-PCR
FlS1-QF	CCCCTCTCGCATCAACTACC	qRT-PCR
FlS1-QR	CCCACCGCCTCTTTGAGAAT	qRT-PCR
FlS2-QF	CTGGATCGACGCCAAGTACG	qRT-PCR
FlS2-QR	TGGGTCCCACTACAAACTCC	qRT-PCR
CHI2-QF	TCTGTTTCTTGGCGGCGCAG	qRT-PCR
CHI2-QR	ACCCGGCGGGAAATTCTGAT	qRT-PCR
F3H-VGS-F	GCTCTAGACTACAACGAATTCAGCACCGA	VIGS
F3H-VGS-R	CGGGATCCGCGGGTACGAGAAGTATGTC	VIGS
